# Synthetic Biology: Mapping the Scientific Landscape

**DOI:** 10.1371/journal.pone.0034368

**Published:** 2012-04-23

**Authors:** Paul Oldham, Stephen Hall, Geoff Burton

**Affiliations:** 1 ESRC Centre for Economic and Social Aspects of Genomics (Cesagen), Lancaster University, Lancaster, United Kingdom; 2 Institute of Advanced Studies (UNU-IAS), United Nations University, Yokohama, Japan; Argonne National Laboratory, United States of America

## Abstract

This article uses data from Thomson Reuters *Web of Science* to map and analyse the scientific landscape for synthetic biology. The article draws on recent advances in data visualisation and analytics with the aim of informing upcoming international policy debates on the governance of synthetic biology by the Subsidiary Body on Scientific, Technical and Technological Advice (SBSTTA) of the United Nations Convention on Biological Diversity. We use mapping techniques to identify how synthetic biology can best be understood and the range of institutions, researchers and funding agencies involved. Debates under the Convention are likely to focus on a possible moratorium on the field release of synthetic organisms, cells or genomes. Based on the empirical evidence we propose that guidance could be provided to funding agencies to respect the letter and spirit of the Convention on Biological Diversity in making research investments. Building on the recommendations of the United States Presidential Commission for the Study of Bioethical Issues we demonstrate that it is possible to promote independent and transparent monitoring of developments in synthetic biology using modern information tools. In particular, public and policy understanding and engagement with synthetic biology can be enhanced through the use of online interactive tools. As a step forward in this process we make existing data on the scientific literature on synthetic biology available in an online interactive workbook so that researchers, policy makers and civil society can explore the data and draw conclusions for themselves.

## Introduction

Synthetic biology is a growing focus of scientific and public policy attention with respect to safety [1]–[3], security [4], [5], ethics [6]–[8], intellectual property [9]–[12] and the potential benefits or negative impacts of this emerging field. Pioneering work by NGOs such as the ETC Group and headlines announcing the creation of artificial life have increasingly brought policy attention to bear on synthetic biology [13]. In April of 2012 the Subsidiary Body on Scientific, Technical and Technological Advice (SBSTTA) of the United Nations Convention on Biological Diversity will consider the potential implications of synthetic biology as a new and emerging issue. Recommendations from SBSTTA will then go forward to the Eleventh meeting of the governing Conference of the Parties (COP11) in India in October 2012 for a decision.

Parties to the Convention on Biological Diversity will be particularly interested in the potential implications of synthetic biology for the three objectives of the convention: the conservation of biodiversity; the sustainable use of biodiversity, and; the fair and equitable sharing of benefits arising from the utilization of genetic resources. SBSTTA and the Parties to the Convention are likely to focus on the potential implications of the field release of synthetic organisms, cells or genomes into the environment for biodiversity in light of the objectives of the Convention and the precautionary approach (decision X/13 para. 4).

This article aims to inform upcoming debates on the governance of synthetic biology by establishing a baseline for mapping the core of the scientific landscape for synthetic biology. We build on two recent advances in visualisation and interaction with scientific information. The first is visualisations of networks of words, organizations, authors and funding bodies using the open source network mapping tool, Gephi. The second is the use of Tableau analytics software to provide interactive visualisation of information on publications about synthetic biology from Thomson Reuters *Web of Science*.

The effect of these approaches is to improve the overall transparency of synthetic biology to researchers, policy-makers and civil society interested in the emergence of synthetic biology. The growing availability of digital data and analytical tools means that the critical links between data and analysis in social scientific contributions to evidence based debates can be maintained and presented in new ways. The emergence of these tools opens up data to allow researchers, policy makers and civil society to explore data for themselves, to raise their own questions and draw their own conclusions. The data in this article is available as an interactive Tableau workbook in Workbook S1 for use with free Tableau Reader software and online through the Synthetic Biology Scientific Landscape Tableau Public workbook (Legends S1).

## Methods

In approaching a new and emerging area of science and technology within the scientific literature a variety of search strategies may be used with Thomson Reuters or other publication databases. The choices made in the development of search strategies affect the number and nature of the results. Thus, searches of the Thomson Reuters *Web of Knowledge* capture a wide range of results across disciplines and publication sources but are very limited in terms of data fields for analysis. In contrast, Thomson Reuters *Web of Science* provides a narrower spectrum of results from three major scientific indexes and conference proceedings but includes all major data fields for analysis. We selected *Web of Science* because of the large range of fields available for text mining and analysis using software tools such as Vantage Point from Search Technology Inc. All databases suffer from lag times between the publication of articles and their appearance in databases.

The second important methodological issue is the use of search terms. We engaged in experimentation with a range of search terms using corpus linguistics approaches and Natural Language Processing to identify individual words and phrases of relevance from samples of articles from *Web of Science* and reports on synthetic biology. This revealed that, in the absence of a controlled vocabulary, the terms used in synthetic biology such as biotechnology or protein engineering are rapidly swamped by uses of the same terms in other research areas. While it is tempting to use ever more refined terms we came to the conclusion that synthetic biology is a self-defining community of researchers from a variety of disciplines who are articulating themselves around the term synthetic biology and related terms such as synthetic genomics. For this reason a simple search strategy focusing on “synthetic biology”, “synthetic genomics”, “synthetic genome” and “synthetic genomes” was used to capture the core landscape. Other important contributions to the field are captured through analysis of cited literature and exploration of the landscape of researchers citing work in synthetic biology within their publications (below).

Searches were conducted using the topic field in *Web of Science* which encompasses the title, abstract, author keywords and terms appearing in the title of cited literature (keywords plus). The results were then imported into Vantage Point software from Search Technology Inc. for text mining and processing of geographical, institutional and funding data fields. Publications citing this core landscape were also selected in *Web of Science* to exclude self-citations, deduplicated and processed in Vantage Point. The processed data was then geocoded using Yahoo Place Finder and exported for visualisation and geographical mapping in Tableau Desktop and Gephi network mapping software. Data was prepared for display in Tableau Public by splitting relevant fields to extract values (i.e. country names). Gephi visualisations deployed the Fruchterman-Reingold and Force Atlas algorithms with nodes manually adjusted to ensure label clarity. The data presented in this article is limited to the literature available in *Web of Science* at the time searches were conducted in early January 2012. Data on literature citing the core landscape for synthetic biology is limited to the 5,995 deduplicated citing publications available in *Web of Science* at the time of search.

## Results

### The Rise of Synthetic Biology

**Figure 1:**
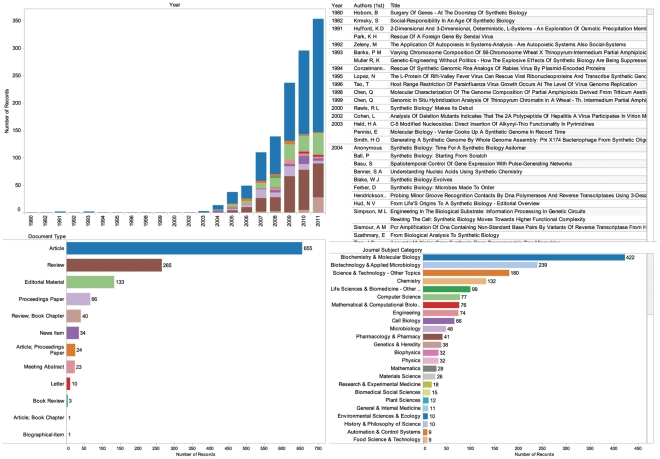
Publication Trends. Data from *Web of Science* topic search for synthetic biology or synthetic genomics or synthetic genome or synthetic genomes in January 2012. Data for recent years may be partial due to lag times. This data can be explored in Workbook S1.

As of January 2012 a total of 1,255 publications were listed in *Web of Science* for synthetic biology and synthetic genomics in the period to the end of December 2011 (Figure 1). These results include publications and conference proceedings produced by researchers active in the development of synthetic biology and work by social scientists and others concerned with understanding the implications of synthetic biology. This data can be explored in Workbook S1 and online through the Synthetic Biology Scientific Landscape.

Viewed historically, references to synthetic biology appeared sporadically in the literature in the early 1980s and 1990s [14]–[18]. These early works included recognition of the historical legacy of Leduc’s (1912) *La Biologie Synthétique* [17] while work by Krimsky in 1982 anticipated much of the recent debates on the social, ethical and economic implications of synthetic biology [15].

Following this brief flurry the record largely fell silent with the exception of work in relation to rescuing synthetic genomic RNA analogs from the rabies virus by Conzelmann et al and on the L-protein of rift valley virus and the transcription of synthetic genome-like RNA molecules by Bouloy et al during the mid-1990s [19], [20]. In the year 2000 Rawls featured work by Eric Kool and declared the “debut” of synthetic biology [21]–[23]. However, it was only in 2007 that the number of publications, excluding conference proceedings and news items, exceeded 100 records. A significant proportion of the literature takes the form of review articles with a provisional total of 62 review articles against 207 articles in 2011 (Figure 1). This profile of review against research articles suggests an emerging field. Compared with the emergence of nanotechnology, which records a basic 15,924 publications in *Web of Science* for the same period, synthetic biology remains small scale.

Defining or characterising synthetic biology has become a significant focus of discussion among researchers [24]–[30]. Three characterisations of synthetic biology provide an insight into these debates. Benner and Sismour identify two broad classes of synthetic biologists [25]. The first class focus on assembling non-natural or synthetic components to create chemical systems that support Darwinian or biological evolution. The second class are informed by engineering and focus on extracting interchangeable parts from living systems to create construction units and devices that may or may not be analogous with existing biological systems (Benner and Sismour 2005: 553) Both classes focus on the chemical synthesis of biological components ranging from gene circuits to entire genomes. However, the first class is concerned with understanding ‘natural’ biology while the latter focuses on engineering.

Endy subdivides synthetic biologists into four main groups: biologists, chemists, ‘re-writers’ and engineers [26]. For biologists, synthetic biology provides a means to understand natural biological systems. For chemists it is an extension of synthetic chemistry leading to the development of novel molecules and advancing research on the origin of life. For ‘re-writers’ synthetic biology offers the promise of optimising biological systems including ‘refactoring’ existing genomes [30]. Finally, for engineers biology is classified as a ‘technology’ that requires “the development of foundational technologies that make the design and construction of engineered biological systems easier” (Endy 2005: 449).

De Lorenzo and Danchin describe synthetic biology as an “inclusive theoretical and technical framework in which to approach biological systems with the conceptual tools and language imported from electrical circuitry and mechanical manufacturing” to pursue “the rational combination of standardised biological parts that are decoupled from their natural context” [31]. From their perspective “The fundamental idea behind synthetic biology is that any biological system can be regarded as a combination of individual functional elements - not unlike those found in man-made devices. These can therefore be described as a limited number of parts that can be combined in novel configurations to modify existing properties or to create new ones” (De Lorenzo and Danchin 2008: 822).

The emergence of synthetic biology has been accompanied by calls for independent evaluation and monitoring of this field, notably by the 2010 report of the United States Presidential Commission for the Study of Bioethical Issues [8]. Scientometrics methods can contribute to such assessments by focusing on basic questions of who, what and where in an empirically rigorous manner. We turn first to the exploration of the language used in synthetic biology to describe this field.

### The Language of Synthetic Biology

**Figure 2:**
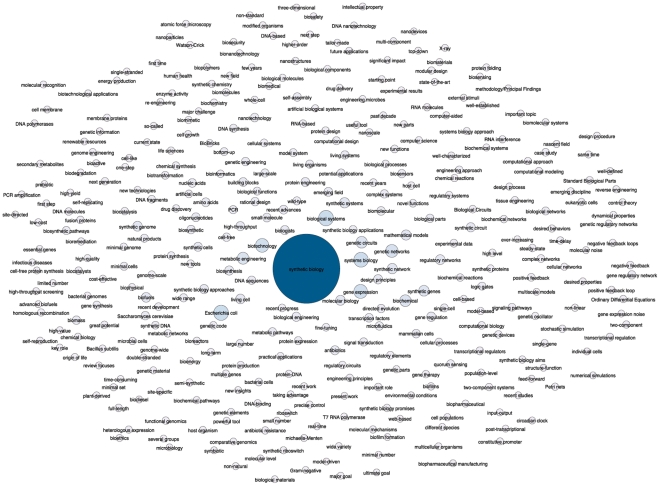
Key Terms Network. A Fruchterman-Reingold representation in Gephi of the top 356 aggregated terms of 36,262 terms within *Web of Science* literature for synthetic biology based on titles, abstracts and author keywords following stemming. Node size is based on the number of records. Node positions have been adjusted to clarify labels.

Scientometrics approaches typically use key words and phrases to explore emerging areas of science [32], [33]. A total of 36,262 individual words and phrases from the titles, abstracts and author key words of publications were available for analysis from our dataset of 1,255 publications. These terms were then reduced to 24,023 multi-word phrases and composite terms (i.e. biotechnology, bionanotechnology) to focus on meaningful concepts and categories. The terms were then grouped using word stemming to capture variations of leading terms i.e. biological systems AND artificial biological systems or metabolic engineering AND metabolic pathway engineering. This method revealed that 356 terms capture 99% of records on synthetic biology. As we might expect, the top unifying term is synthetic biology. When synthetic biology was excluded the remaining 355 terms captured 88% of records providing sufficient accuracy for representation of the data. The 355 terms were then placed in a co-occurrence matrix that provides a quantitative measure of the number of records for each term (nodes) and the strength of connections between terms (edges). These relationships were then visualised using the open source Gephi network analysis software (Figure 2).

Figure 2 reveals that synthetic biology is concerned with biological systems using approaches from systems biology directed to biotechnology that involves gene expression, gene networks, metabolic engineering and genetic circuits, synthetic genes and synthetic networks. As we move from central terms to the outer periphery of the network less frequent terms such as biosafety, bioethics, and intellectual property emerge to reveal the wider spectrum of issues revolving around the core of synthetic biology.

**Figure 3:**
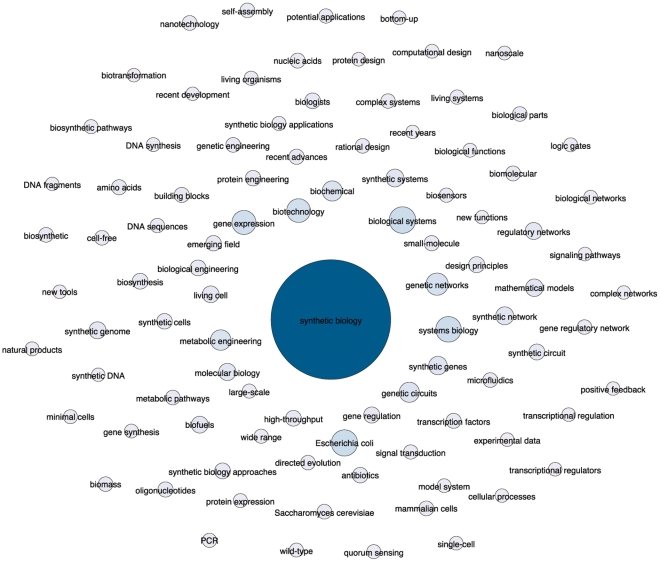
Top Terms. A Fruchterman-Reingold representation in Gephi of the top aggregated terms for synthetic biology within *Web of Science* based on titles, abstracts and author keywords appearing in more than 20 or more records following stemming. Node size is based on the number of records. Node positions have been adjusted to clarify labels.

Figure 3 narrows the focus to the top ranking phrases and composite terms in more than 20 records across the dataset. In considering these results it may be tempting to widen the search criteria for synthetic biology to include additional top occurring key terms to enhance data capture. For example, synthetic biology is strongly associated with systems biology, protein engineering, genetic engineering and nanotechnology. However, synthetic biology would immediately be swamped by the results from these much larger fields. In practice, synthetic biology is being constructed from a combination of convergences and overlaps with other areas of science and technology, some of which, such as metabolic engineering, are new and emerging areas of research.

Our understanding of the distinctiveness of synthetic biology is improved by removing dominant terms to expose underlying networks. This approach revealed the prominence of relations between genetic circuits, gene networks, synthetic genes, synthetic systems and gene regulatory networks [34], [35]. Viewed from this perspective synthetic biology is being constructed from a core of work around genetic circuits and networks. Much attention has understandably focused on the potential implications of synthetic cells, genomes and organisms. However, the creation of synthetic circuits, synthetic genes, and synthetic gene networks may eventually be more likely to find routine expression in organisms and make their way into the wider environment.

It is also important to note the incipient diversification of synthetic biology. This is apparent in the case of mammalian synthetic biology [36], cell free synthetic biology [37] and chemical synthetic biology [38]. Genome engineering [39] can be classified alongside genome-scale synthetic biology [40] and work in synthetic genomics to create synthetic genomes as popularised by the J. Craig Venter Institute. Other emerging variants of synthetic biology include in-vitro synthetic biology [41], RNA synthetic biology [42], cyanobacterial synthetic biology [43], plant synthetic biology [44] and nano-enabled synthetic biology [45]. While low in frequency, these modules or flavours of synthetic biology suggest the diversification and potential fragmentation of the field. This is important for policy debates because the longer term implication is that synthetic biology may cease to be a ‘unitary’ object for policy action and become multiple in applications to particular organisms or the components of organisms.

Viewed purely from the perspective of key terms, synthetic biology emerges as a research mobilisation around the term “synthetic biology” and, to a lesser extent, “synthetic genomics” that draws on methods, techniques and technologies from a wider range of established and emergent research areas. That is, synthetic biology is a rallying flag around which researchers are articulating themselves focusing on genetic circuits, networks, pathways and parts and extending to minimal cells, genome transplantation, synthetic genomes and whole genome engineering. Synthetic biology draws on a variety of techniques from systems biology, metabolic engineering, protein engineering and genetic engineering but cannot simply be reduced to these fields. Even as synthetic biology emerges as a rallying flag around which researchers are articulating themselves it is also diversifying and, at least potentially, fragmenting into specialist areas focusing on particular approaches and classes of organism. As we will see below in exploring the citing landscape the impacts of synthetic biology are also being disseminated and picked up in multiple other fields.

### Networks and Impacts

**Figure 4:**
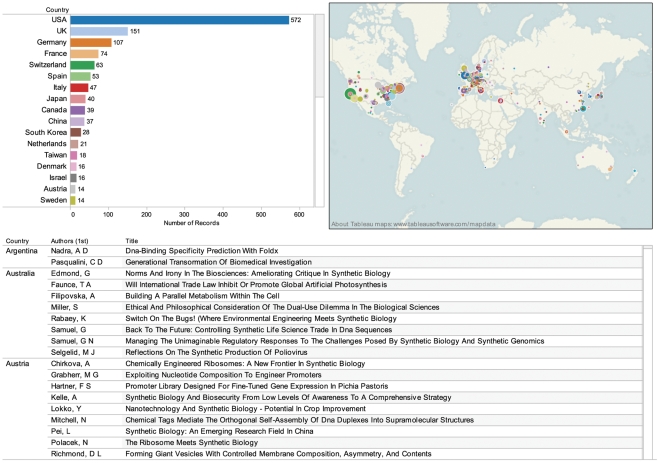
Country Rankings and Organization Distribution Map. The figure ranks countries on the number of authors from a country appearing in publications in *Web of Science* linked to geocoded organizational information on their global distribution. Country rankings are base on data for 1160 records of 1255 records. This data can be explored in Workbook S1.

In total 40 countries are involved in the core landscape for research on synthetic biology. Figure 4 displays the rankings and locations of these countries and organizations. This data can be explored in Workbook S1 and online through the Synthetic Biology Scientific Landscape. *Web of Science* data reveals that synthetic biology is dominated by the United States followed by the UK, Germany, France and Switzerland. Emerging major economies, notably China, Brazil, and India, along with Mexico, Argentina, South Africa and Singapore are also appearing in the core scientific landscape.

**Figure 5:**
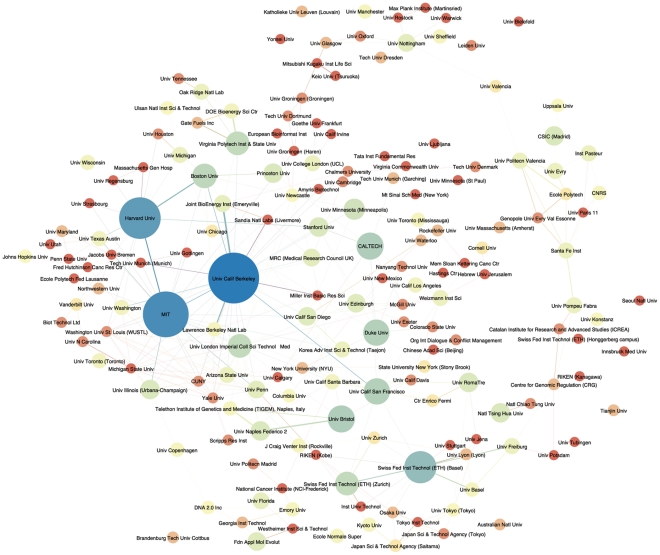
Organization Network. This network map shows all author organizations with more than three publication records in *Web of Science* for the core landscape for synthetic biology. Where available this network map distinguishes organizations by locations (i.e. ETH) to distinguish groups. This is a Fruchterman-Reingold algorithm network representation in Gephi. The original dense representation was expanded and nodes were manually adjusted to prevent label overlaps and reduce irrelevant node to edge intersections.

The emergence of networks of countries reflects the underlying growth of international collaborative research networks between institutions and research groups. Mapping of institutions and organizations revealed 682 organizations with offices in various locations around the world. Figure 5 provides a visualisation of the network of organizations with three or more records in *Web of Science* data.

In terms of the volume of publications network mapping reveals the prominence of the University of California at Berkeley, the Swiss Federal Institute of Technology (ETH), Harvard and MIT. The data does not discriminate by discipline with the social sciences represented in work at Berkeley, Exeter and Edinburgh while law is represented in work at Duke University [7], [10], [46], [47].

In practical and policy terms, this data informs us that any regulatory measures that apply to synthetic biology will primarily be targeted at the 682 organizations in 40 countries within this network and any new organizations and countries that subsequently join the network. As we will see below, research results in this field are increasingly being picked up by other researchers resulting in the expansion of the number of countries and organizations involved in, or influenced by, research in synthetic biology. Mapping of the scientific literature provides a basis for engaging in dialogue with the spectrum of researchers and institutions engaged in research on synthetic biology and for monitoring synthetic biology over the long term using empirical evidence.

**Figure 6:**
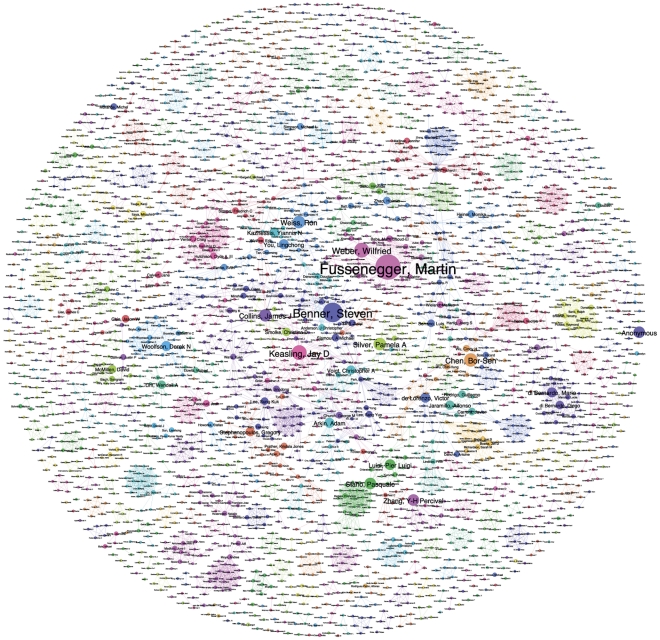
Author Network. This network map shows the relationships between 527 clusters of authors with publications on synthetic biology in *Web of Science*. Node size is based on the number of publications for a given author.

**Figure 7:**
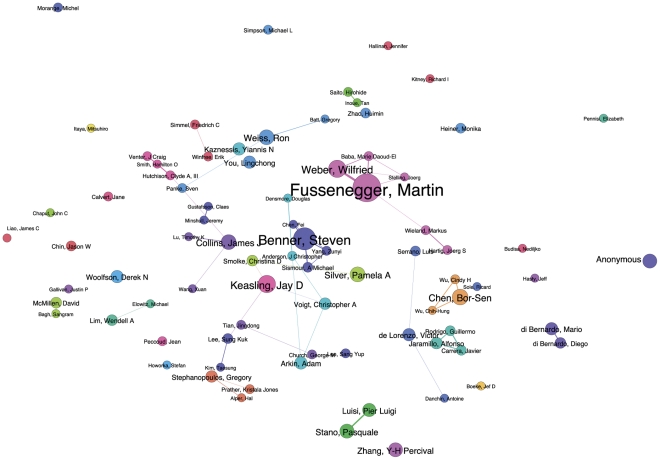
Main Authors. This network map shows the network of authors with five or more publications on synthetic biology in *Web of Science*. Node size is based on the number of publications for a given author. Node position has been manually adjusted from Figure 5 to clarify labels.

In practice, inter-institutional collaborations are embodied in individual researchers and research groups. In total, 2,934 authors were identified in the available data from *Web of Science* for the core landscape. Network mapping in Gephi using the modularity class algorithm revealed 527 distinct research clusters or modules that make up the primary human resources for synthetic biology (Figure 6) [48]. Ranking authors purely by the number of publications revealed the leading authors to be Fussenegger [49], Benner [25], Keasling [50], Weber [51], Chen [52], Collins [24], Silver [53], Weiss [54], Stano [55] and Zhang [56]. The details of the network come into greater focus in Figure 7 that ranks all authors with 5 or more publications within the data. This data includes one social scientist [46] to capture the wider network of those working on and writing about synthetic biology.

The citation of articles within the wider scientific literature can provide important insights into research that is shaping an emerging field and into the impacts of research in a particular field. Researchers themselves are familiar with citation scores as an indicator of prestige and they can be important for career progression in some disciplines. However, citation scores need to be approached with considerable caution. Citation scores are heavily biased towards journal publications and publication and citation practices vary significantly across disciplines such as biology, computer science, mathematics and engineering [57]. This produces problems in assessing the importance of literature in the case of interdisciplinary research [58]. Furthermore, access to citation data may be limited and lacking in transparency.

Taking these difficulties into account we avoid a ‘top cited’ style analysis and seek to illuminate the scientific landscape for synthetic biology in two ways. First, by examining the literature actually cited by researchers inside the core landscape. Second, by exploring the wider landscape of literature citing the core landscape.

The literature cited by participants in a particular research community provides important insights into research that is shaping a field. In the case of the core landscape approximately 25,567 authors appear in 37,217 cited references. Unfortunately, cited references are only available in raw form i.e. Elowitz MB, 2000, NATURE, V403, P335. This is compounded by a requirement to retrieve cited references individually in *Web of Science*. As such, it is not presently realistic to fully map and explore the cited literature within synthetic biology.

**Figure 8:**
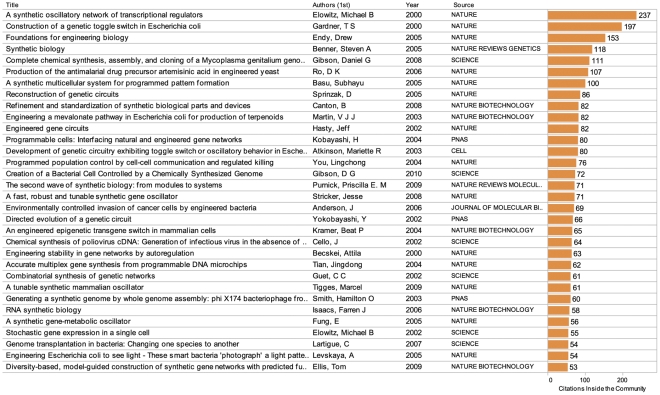
Key Articles in the Core Landscape. This figure shows the main articles cited by other authors inside the core landscape for synthetic biology. Data is based on counts of citations in the cited literature field of publications in the core landscape. The data does not refer to total citations for an author or article within the wider scientific literature. This data can be explored in Workbook S1.

What can be achieved is an insight into the cited literature. Figure 8 provides a summary of the top 30 references cited inside the core landscape. This data is accessible in Workbook S1 and online through the Synthetic Biology Scientific Landscape. The top 5 publications cited inside this community are work by Elowitz [34] on transcriptional regulation, Gardner [59] on a toggle switch in *E. coli*, Gibson et. al [60] on the complete chemical synthesis of the *Mycoplasma genitalium* genome, Endy [26] on engineering biology and Benner and Sismour [25] on synthetic biology. This data can also be explored for all authors with five or more citations in the core landscape in Workbook S1 and online through the Synthetic Biology Scientific Landscape. Future research could usefully focus on exploring the cited references to examine other important articles in this field. The data presented here is a first step in that process.

An insight into the wider impacts of existing research in synthetic biology is provided by the literature citing publications in the core landscape. Data on citing publications from the core landscape was generated using the Citation Report function within *Web of Science*. After excluding self-citations and publications in the core dataset we identified 5,955 distinct citing publications available in *Web of Science*. Trends within this wider landscape can be explored in Workbook S1 and online through the Synthetic Biology Scientific Landscape.

The citing literature reveals an expanded landscape involving 78 countries, approximately 3,000 organizations, and an estimated 19,751 researchers. In total 1,153 of the 2,934 researchers working on synthetic biology are present in this landscape and dominate the publication rankings. Of particular importance within this landscape, in terms of the number of publications, is work by Keasling on biofuels [61], Kell on systems biology and the reconstruction of a yeast metabolic network [62], Baric in relation to the GII.4 norovirus and related work on the SARS virus [63], work by Conzelmann on the rabies virus and gene therapy [64], Fussenegger on gene therapy [65], and Nielsen on systems biology in areas such as antibiotic production by microorganisms [66].

The citing landscape also reveals other authors working outside the community of researchers writing on synthetic biology that demonstrates both the impact and diversification of the influence of this field. Notable here is work by Katz on biocomputing [67], [68], Dorrestein on the biosynthetic origin of natural products from marine microorganisms and on multiplex sequencing of peptide antibiotics [69], [70], along with work by Flick on reverse genetics of negative stranded RNA viruses including research on Crimean-Congo hemorrhagic fever virus and Rift Valley fever virus [71]–[73]. Looking beyond work directed towards potential applications, research by Dunn focusing on metabolomics and mass spectrometry provides a reminder of the importance of methodological development [74], [75].

When viewed from the perspective of the language a defining characteristic of the citing landscape is the absence of references to synthetic biology, synthetic genomics and synthetic genomes. In the absence of such unifying terms, the citing landscape is diffuse and characterised by low frequency terms relative to the size of the landscape. However, at the apex of this landscape we find concentrations in work on E. coli by researchers in synthetic biology such as Collins [76], along with the prominence of terms such as gene expression [77], systems biology [78] and metabolic engineering [79].

The citing landscape is also important as an indicator of knowledge transfer between countries and the emergence of research groups informed by work in synthetic biology. The citing landscape encompasses researchers from 78 countries. What is immediately apparent is that countries such as China have risen from 10^th^ place in the core landscape to 4^th^ place and India has risen from 18^th^ to 16^th^ place while Brazil has also risen in the rankings. What is less apparent is that researchers from the Africa region are also beginning to appear in the literature. Examples of this development include Egypt for chemoenzymatic and microbial dynamic kinetic resolutions [80], Ghana on the SARS coronavirus in bats [81], Nigeria in the case of an ethnobotanical survey and cytotoxicity testing in plants for potential cancer treatments [82] and South Africa in research on synthetic promoters and genetic control through cis engineering [83]. Data by countries within the citing landscape can be explored in Workbook S1 and online through the Synthetic Biology Scientific Landscape.

**Figure 9:**
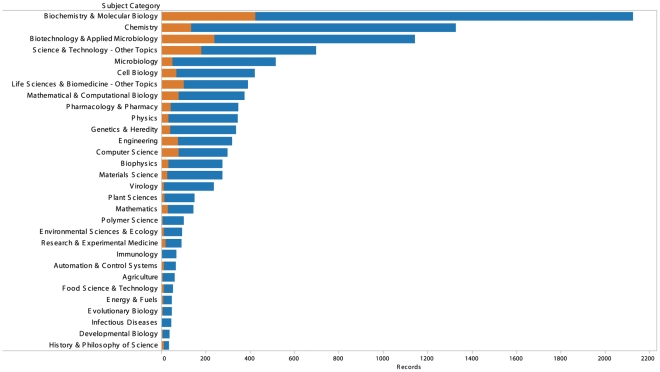
Journal Subject Categories Core and Citing Landscapes. This figure compares the Thomson Reuters Web of Science journal subject categories for journal articles in the core landscape (orange) with those in the citing landscape (blue). Data was split on the subject category field to focus on individual journal subject categories.

We are also witnessing the influence of research in synthetic biology within other fields. In the case of the journal literature, a quantitative insight into this influence is provided by comparing Thomson Reuters *Web of Science* journal subject categories from the core and the citing landscape (Figure 9). Figure 9 makes clear that the core of synthetic biology is strongly situated in Biochemistry & Molecular Biology, followed by Biotechnology & Applied Microbiology and the interdisciplinary Science & Technology category. In the citing landscape these categories are reinforced and shift in importance notably through the influence of synthetic biology in chemistry. The impacts of synthetic biology are also observed in the emergence of publications in Virology, Environmental Sciences and Ecology, for subjects such as bioremediation, and Immunology focusing on areas such as antibiotics, biofilms and vaccine. Data on journal subject categories for the citing landscape can be explored in Workbook S1 and online through the Synthetic Biology Scientific Landscape. Future work in exploring the emerging scientific landscape for synthetic biology could build upon recent efforts to map the structure of the scientific literature using journal subject categories and situate synthetic biology within this structure [84]–[86].

The preceding analysis of the emerging networks and impacts of synthetic biology within the scientific literature has demonstrated the increasing internationalisation of synthetic biology and its dissemination across a range of disciplines and research areas. We now turn to analysis of the network of funding organizations involved in supporting the core landscape for synthetic biology.

### Funding

**Figure 10:**
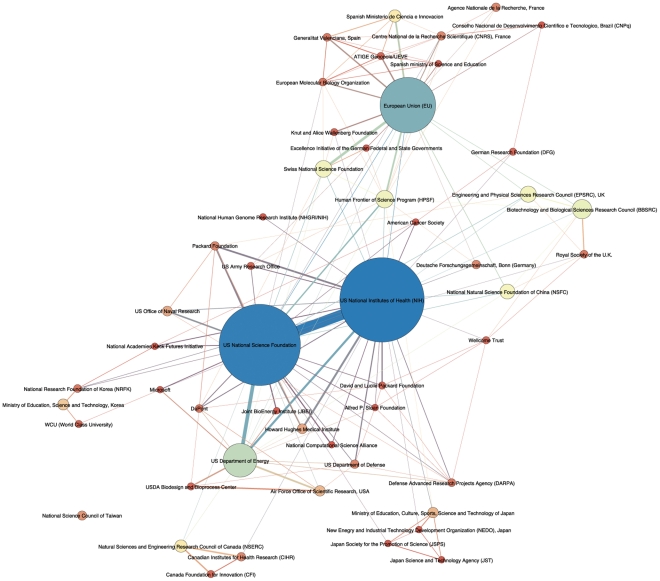
Top Funding Organizations. This map shows the network of relations between funding organizations appearing in 5 or more publications within *Web of Science* on synthetic biology. The figure was generated using the Force Atlas algorithm and the results were manually adjusted with the objective of preventing incorrect node to edge intersections. It should be noted that large nodes such as the US National Science Foundation may display intersections with unrelated edges.

One of the potential opportunities to introduce appropriate governance measures is provided by focusing on the institutions and organizations that fund synthetic biology. Until recently, data on funding for scientific research was largely obscured in publication data. *Web of Science* now includes limited information on sources of funding. For synthetic biology data is limited to 562 records representing 44% of records in our dataset published from 2007. Furthermore, data is restricted to information in *Web of Science,* lacks standards of description, and does not provide an insight into the size of awards. However, it is possible to gain a partial insight into the nature of funding organizations and emerging networks of funding organizations. After cleaning the raw data we identified approximately 530 organizations as funding sources for synthetic biology. Figure 10 provides a visualisation of the top funding organizations that have supported research appearing in three or more publications in *Web of Science*.

Funding is dominated by the National Institutes of Health (NIH) and the National Science Foundation (NSF) in the United States and the European Union Framework programme, followed by the US Department of Energy, the combined agencies of the US Department of Defense, the Biotechnology and Biological Sciences Research Council in the UK (BBSRC), the Human Frontier Science Foundation (HPSF), and the Swiss National Science Foundation. The Human Frontier Science Program based in Strasbourg is an international programme established by Australia, Canada, France, Germany, India, Italy, Japan, South Korea, Norway, New Zealand, Switzerland, the UK, the European Union and the United States. The UK would rise in the rankings measured on publications if its sister organizations (the Engineering and Physical Sciences Research Council or EPSRC) and the combined Research Councils UK were aggregated.

The NIH has funded notable research on synthetic gene oscillators [87], an ER mitochondria screening tethering complex using a synthetic biology screen [88], work on synthetic genetic networks [89] and development of the proposed second wave of synthetic biology in moving from modules to systems [27]. The National Science Foundation has funded research focusing on metabolic engineering for biofuel production [90]–[93], a synthetic platform organism for biotechnological applications [94] and biodegradation pathways [95]. European Union research funding has supported work on bistability and epigenetic inheritance and bet hedging in bacteria [96], a yeast synthetic network for assessing the outcomes of reverse engineering and modelling [97] and bioremediation [98]. The US Department of Energy, as is well known from the work of the J. Craig Venter Institute, has been funding research on biofuels [91], [99] along with work on a synchronized genetic clock for engineering genetic circuits [100] and whole genome engineering [39]. The United States Department of Defense, through the Defense Advanced Research Projects Agency (DARPA), the Office of Naval Research, the Air Force and the Army, emerges as an important co-funder of research with other agencies in areas such as bacterial quorum sensing with respect to biofilms and disease [27], [87], [101].

Funding by the Swiss National Science Foundation appears to be more targeted towards biopharmaceutical applications and work relating to mammals [36], [51], [102]–[104]. This highlights that research funders may begin to focus on distinctive emerging flavours of synthetic biology. In the UK the BBSRC has funded work reviewing synthetic biology [105] has served as a co-funder for work on bistability and epigenetics [96], designing and encoding models for synthetic biology and engineering protein assemblies [106], [107].

Companies are represented in the data principally through DuPont in work on genetic circuits [108], [109] and renewable carbohydrates as hydrogen carriers [110]. In computing Microsoft has provided research funding for software and bioinformatics related research and teaching in these fields [111], [112] while IBM has contributed to work in metabolic engineering [113]. Mathworks, a US company specialising in mathematical computing software, also emerges as a sponsor of work on gene circuits [114]. Specialist synthetic biology companies such as LS9 emerge as sponsors of work on microbial fuels [115] as well as the well known work of Synthetic Genomics through the J. Craig Venter Institute. Large pharmaceutical companies are represented by Pfizer in research on PCR-less library mutagenesis [116] and Roche in the development of the SynBioWave software suite [117]. Looking outside this data Novartis is present as a funder of research through work on vaccine development that links to collaborations with the J. Craig Venter Institute and Synthetic Genomics [118].

Data by funding organization can be explored in Workbook S1 and online through the Synthetic Biology Scientific Landscape. This type of information is rarely made visible and funding networks may not be visible to the agencies engaged in supporting research. We would emphasise that data on the funding network is limited to 44% of available records and further work is desirable to standardise funding data in future research. Furthermore, this data is unlikely to fully or adequately reflect private sector involvement. A fuller picture will be generated by ongoing work to map the patent landscape. However, the predominance of organizations funded by taxpayers within the scientific literature provides important potential levers for policy makers under the Convention on Biological Diversity seeking to promote respect for the letter and spirit of the Convention.

## Discussion

193 governments are Parties to the Convention on Biological Diversity. Additional countries, notably the United States, are signatories but have not yet ratified the Convention. The rise of synthetic biology is of relevance to the three objectives of the Convention concerning the conservation, sustainable use and fair and equitable benefit sharing arising from the utilization of genetic resources. Synthetic biology is also relevant to the protocols established under the Convention with respect to biosafety, liability and redress, and access to genetic resources and benefit sharing. We briefly address each of these areas before considering proposals for a moratorium on the field release of synthetic organisms, cells and components.

163 countries are Parties to the Cartagena Protocol on Biosafety under the Convention that is concerned with regulation, risk assessment and liability issues for the movement of Living Modified Organisms (LMOs). Under Article 3(g) of the Cartagena Protocol a living modified organism “means any living organism that possesses a novel combination of genetic material obtained through the use of modern biotechnology.” Article 3(i) defines modern biotechnology as: “a) in vitro nucleic acid techniques, including recombination deoxyribonucleic acid (DNA) and direct injection of nucleic acid into cells or organelles, or b) Fusion of cells beyond the taxonomic family that overcome natural physiological reproductive or recombination barriers and that are not techniques used in traditional breeding and selection.” The Cartagena Protocol establishes an advanced informed agreement procedure to provide countries with a basis for making informed decisions on whether to accept shipments of LMOs meeting the above criteria.

In October 2010 the Tenth Conference of the Parties adopted the Nagoya-Kuala Lumpur Supplementary Protocol on Liability and Redress to the Cartagena Protocol on Biosafety. This protocol establishes international rules and procedures on liability and redress relating to living modified organisms. These rules cover situations involving damage arising from transboundary movements of LMOs intended for direct use as food or feed, for what is called “contained use” (i.e. in industrial facilities) and intentional introduction into the environment. The rules also address damage arising from authorized use of LMOs and unintentional and illegal transboundary movements.

The provisions of the Cartagena Protocol are likely to lead to legal questions on whether the products of synthetic biology fall within the scope of the Cartagena Protocol i.e. do the criteria apply only to *in vitro* techniques or approaches involving cell fusion outside the taxonomic family that overcome natural reproductive or recombination barriers as set out in the Cartagena Protocol? Furthermore, it may be that the Cartagena protocol only applies to the physical transfer of LMOs and does not apply to material or digital transfers of genetic sequences, components and parts that may be later used to constitute an LMO [119]. Additional doubts have also been expressed about whether the waiver on the requirement for advanced informed agreement for transfers of LMOs destined for contained use in a facility under Article 6.2 of the Cartagena protocol should apply in the case of synthetic organisms [119]. In the case of the Kuala Lumpur supplementary protocol, this is limited in three ways. First, the rules apply only to transboundary movements that occur after the supplementary protocol has entered into force. Second, the rules are restricted to damage occurring in areas under national jurisdiction (i.e. excluding the high seas and treaty areas such as Antarctica). Third, in the case of damage arising from transboundary movements of LMOs from countries that are not party to the supplementary protocol then Parties are only able to apply domestic implementing legislation for the protocol, rather than seeking redress through an international body.

Synthetic biology is also relevant to the third objective of the Convention on access to genetic resources and benefit sharing and the recently concluded Nagoya Protocol on Access to Genetic Resources and the Fair and Equitable Sharing of Benefits Arising from their Utilization. Here concerns have been expressed with regard to the use of artemisinin and the increasing ease with which genetic material can be transformed into digital information, transmitted, reproduced and manipulated [119]. Research on natural product based drug discovery in synthetic biology is closely linked with metabolic engineering [120] and reviving interest in compounds from natural products [121] along with the use of engineered microorganisms for drug development [122], [123]. We have also seen that research in synthetic biology is increasingly informing wider research in the citing landscape on drug discovery, antibiotics and vaccines.

We suggest that more detailed empirical analysis of the implications of synthetic biology for access and benefit sharing is desirable notably with respect to the source of materials and the positive or negative economic implications of synthetic biology for developing countries. Here we note that synthetic biology may provide cost effective means for drug discovery and development. Positive outcomes may be possible for developing countries where drug discovery focuses on neglected diseases. The emerging involvement of researchers from China, India, Brazil, Mexico and South Africa in work on synthetic biology may provide positive opportunities for funding bodies to promote research directed to the needs of populations in developing countries. As part of this process funding bodies could also be encouraged by Parties to the Convention to contribute to the effective implementation of the Nagoya Protocol on Access to Genetic Resources and Benefit Sharing as the new international standard governing access and benefit-sharing for genetic resources across a spectrum of research fields.

The main focus of debate at SBSTTA and the 11^th^ Conference of the Parties to the Convention on Biological Diversity will be the potential field release of synthetic life, cells or genomes into the environment taking account of the precautionary principle. In preparation for this debate a number of civil society organizations, including the ETC Group, The Center for Food Safety, Econexus, Friends of the Earth USA, the International Center for Technology Assessment and the Sustainability Council of New Zealand have made individual and joint submissions on this topic. The key recommendation by the International Civil Society Working Group on Synthetic Biology (ICSWGSB) is that:

“Parties to the Convention on Biological Diversity, in accordance with the precautionary principle, which is key when dealing with new and emerging scientific and technological issues, should ensure that synthetic genetic parts and living modified organisms produced by synthetic biology are not released into the environment or approved for commercial use until there is an adequate scientific basis on which to justify such activities and due consideration is given to the associated risks for biological diversity, also including socio-economic risks and risks to the environment, human health, livelihoods, culture and traditional knowledge, practices and innovations” (ICSWGSB 2011: 5).

These organizations further call upon governments to “submit views and national experiences and identify gaps in the governance of synthetic genetic parts and living modified organisms produced by synthetic biology as developed for release or commercial use” as a basis for further work under the Convention (ICSWGSB 2011: 5). In addition they recommend that countries conduct impact assessments for proposed synthetic biology projects and that in the absence of reliable data on biocontainment strategies “products incorporating such technologies should not be approved by Parties for field testing…” or commercial use until there is adequate scientific data on their environmental and socio-economic impacts (ICSWGSB 2011: 6). If adopted these recommendations would have serious implications for the conduct of scientific research in synthetic biology.

The first of these recommendations constitutes a call for a moratorium on the environmental release of synthetic organisms and synthetic parts and finds a precedent under the Convention in a 2006 decision to introduce a *de facto* moratorium on Genetic Use Restriction Technologies (GURTS or “Terminator” technologies) (decision VIII/23 C) [124], [125]. The specific recommendation on biocontainment appears to reflect concerns about the scientific credibility and implications of proposals that synthetic components or organisms could be engineered to depend on non-natural amino acids [126], or deploy ‘fail fast’ [127] or suicide mechanisms to prevent survival in the natural environment [128].

The question of a potential moratorium on synthetic biology as a field was considered as part of the 2010 United States Presidential Commission for the Study of Bioethical Issues report on synthetic biology [8]. In presenting its report the “PCBSI concluded that synthetic biology is capable of significant but limited achievements posing limited risks. Future developments may raise further objections, but the Commission found no reason to endorse additional federal regulations or a moratorium on work in this field at this time. Instead, the Commission urges monitoring and dialogue between the private and public sectors to achieve open communication and cooperation” (PCBSI 2010: v). In arriving at this conclusion the Commission sought to find a middle ground between a moratorium on synthetic biology pending assessment of all risks and “unfettered freedom for scientific exploration”. The Commission recommended “an ongoing process of prudent vigilance that carefully monitors, identifies and mitigates potential and realized harms over time” (PCBSI 2010: 8). With respect to moral objections, the Commission argued that “Current objections to synthetic biology on moral grounds are often based on concerns regarding activities that the field is currently incapable of carrying out” (PCBSI 2010: 12).

However, while rejecting a moratorium on the field as a whole, the Commission also recognised the potential high risks and uncertainties around the deliberate release of synthesized organisms and recommended an ongoing review “of the ability of synthetic organisms to multiply in the natural environment and identify, as needed, reliable containment and control mechanisms” (PCBSI 2010: 130). In practice, therefore the recommendations from civil society organizations and the US Presidential Commission for the Study of Bioethical Issues, as the major government sponsored review to date, are not incompatible in their main elements focusing on environmental risk and uncertainty. The question becomes the appropriate course of action.

In considering synthetic biology a number of choices will be available to SBSTTA and Parties to the Convention. Thus, SBSTTA may recommend that greater time is taken to receive information about synthetic biology and the implications of potential release of synthetic components and organisms into the environment before a decision is taken on regulating environmental release. It would be open to SBSTTA to include a recommendation to establish a technical expert group to consider the available evidence and to develop detailed recommendations for future consideration by the Conference of the Parties.

However, the Conference of the Parties, as the Convention’s sovereign decision-making body, might decide to introduce a moratorium on the release of synthetic organisms and components based on the existing uncertainties and risks to biological diversity. Such a decision would have major implications for future research in synthetic biology across the spectrum of research areas in which synthetic biologists are involved. The implications for synthetic biologists would need to be balanced against the fundamental importance of biological diversity to human welfare. The question in this case would become whether a balance could be identified which permitted the continued development of synthetic biology as a field while safeguarding biological diversity.

If a moratorium is regarded as desirable by the Conference of the Parties, the design of such a moratorium would merit careful consideration. For example, a moratorium could be designed that provided opportunities for regular periodic review to allow for the development and testing of biocontainment and control strategies. This approach would recognise the presently limited nature of synthetic biology research directed to field release and the limitations of existing research on engineered biocontainment and control strategies. The existence of a moratorium might, as an incidental benefit, send a strong signal to the ‘biohacking’ community on the acceptable limits of behaviour and encourage wider professionalisation in a field involving a meeting of different disciplines and standards. In short, a moratorium could buy time for the field to develop appropriate standards.

Independent of the question of a moratorium, our research reveals that targeting funding organizations provides a key opportunity to promote appropriate governance in synthetic biology. Specifically, Parties could invite funding organizations to ensure that research they fund is supportive of, and does not run counter to, the objectives of the Convention. This is standard language within the text of the Convention. Such an invitation would serve to promote greater awareness of the Convention among public funding bodies and private foundations that recognise the importance of international commitments on the environment. Over the longer term more specific guidance could be developed for funding organizations as understanding of this field improves.

In the intervening period, Parties to the Convention should be encouraged to seek further information on synthetic biology through engagement with the scientific community and be informed by independent scientific assessment of the actual and potential risks to biological diversity posed by synthetic biology. The research presented in this article on the core scientific landscape for synthetic biology provides a basic platform for identifying and engaging with the organizations and researchers involved in synthetic biology and for the development of transparent monitoring mechanisms to inform decision-making.

### Conclusion

This article has aimed to contribute to upcoming debates on synthetic biology under the United Nations Convention on Biological Diversity by mapping the scientific landscape for synthetic biology. Through the exploration of the core landscape of synthetic biology and its impacts within the wider scientific literature we have sought to contribute to the creation of a baseline for wider understanding and engagement with this emerging field. To achieve this objective we have exploited the increasing availability of digital tools for visualisation and interaction with scientific data to promote engagement with this field of research.

Debate under the Convention on Biological Diversity on synthetic biology is likely to focus on the question of a potential moratorium on the field release of synthetic organisms, cells and genomes into the environment. As a contribution to debate on this issue we have established that the core landscape for synthetic biology involves 2,934 researchers from 682 organizations in 40 countries who are supported by a network of approximately 530 funding organizations. These researchers are engaged in work on genetic components, parts and organisms with potential for a wide range of applications. This community has demonstrated a considerable willingness to engage with civil society and policy and to consider appropriate measures for governance. However, at this early stage in the development of this field proposals regarding biocontainment and control remain under developed. In considering the introduction of a possible moratorium on field release Parties will be confronted by the challenge of balancing the fundamental importance of biodiversity to human welfare with recognition of the importance of the “freedom indispensable for scientific research and creative activity” as set out in Article 15.3 of the International Covenant on Economic, Social and Cultural Rights.

In our view, if a moratorium is introduced on field release opportunities should be provided for periodic review of biocontainment and control measures to encourage further development in this area. Furthermore, we have argued that important opportunities exist for introducing appropriate governance measures through the development of guidance for funding bodies. Finally, the core contribution of this article has been to promote the development of longer term monitoring capacity. Taken together we believe that these proposals can help ensure that any action taken under the Convention is balanced and measured and does not unnecessarily impinge on positive developments in research while remaining attentive to the significant potential negative impacts of synthetic biology and the need to ensure they are objectively assessed and addressed.

## Supporting Information

Legends S1 **Explanations for the Tableau Public Synthetic Biology Scientific Landscape online workbook.**
(DOC)

Workbook S1 **Packaged Tableau Workbook for use with Tableau Reader 7.0.**
(ZIP)
