# Supplementary material for: Synthetic Biology: Mapping the Scientific Landscape
Source: PLoS One. 2012 Apr 23;7(4):e34368. doi: 10.1371/journal.pone.0034368 (PMC3335118; doi:10.1371/journal.pone.0034368)
Supplement: Legends S1 — Explanations for the Tableau Public Synthetic Biology Scientific Landscape online workbook. (DOC) [file pone.0034368.s001.doc]

**Legends S1.**

**Explanations for the Synthetic Biology Scientific Landscape Online Worksheets**

This file provides legends for the Tableau Public file [Synthetic Biology Scientific Landscape](http://public.tableausoftware.com/views/SyntheticBiologyScientificLandscape/SyntheticBiologyTrends).

**S1. Synthetic Biology Trends.** This dashboard displays publication trends for synthetic biology within Web of Science, document types, journal subject category and the year, first author and title of the publications.

**S2. Synthetic Biology Countries**. This dashboard shows data from Web of Science for synthetic biology by individual country. The Web of Science author organization, city and country data field has been geocoded using Yahoo! Place Finder to generate a map of organizations involved in research on synthetic biology. The dashboard also displays the first author and title of publications by country. More than one country may be associated with a single record.

**S3. Synthetic Biology Cited Articles.** This dashboard displays the top cited articles appearing in the cited references field of Web of Science data making up the core landscape for synthetic biology. The figure refers to citations of articles inside the core landscape and does not refer to the total number of citations for an individual article.

**S4. Synthetic Biology Explore Cited Authors.** This dashboard shows a selection of authors cited inside the core landscape for synthetic biology and the corresponding citing record within the landscape. The data is organized alphabetically and can be searched by cited author.

**S5. Synthetic Biology Funding.** This dashboard shows the breakdown of Web of Science data on synthetic biology by country and funding organization.

**S6. Synthetic Biology Citing Trends.** This dashboard displays publication trends for the scientific literature from Web of Science that cites the core landscape for synthetic biology. The dashboard displays publication trends, document types, journal subject categories and the year, first author and title of the publications.

**S7. Synthetic Biology Citing Countries.** This dashboard shows data from Web of Science for the citing landscape for synthetic biology by individual country. More than one country may be associated with a single record.

**S8. Synthetic Biology Citing Subjects.** This dashboard shows the journal subject categories from Web of Science for the citing landscape for synthetic biology.
